# Linkage to hepatitis C treatment in two opioid substitution treatment units in Gothenburg, Sweden: a retrospective cohort study

**DOI:** 10.1186/s13011-023-00527-0

**Published:** 2023-03-13

**Authors:** Magdalena Ydreborg, Emil Lundström, Rosanna Kolleby, Sofia Lexén, Elena Pizarro, Jessica Lindgren, Rune Wejstål, Simon B. Larsson

**Affiliations:** 1 Department of Infectious Diseases, Sahlgrenska Academy, University of Gothenburg, Box 480, 405 30 Gothenburg, Sweden; 2 Clinic of Infectious Diseases, Sahlgrenska University Hospital, Region Västra Götaland, Journalvägen, 10 416 50 Gothenburg, Sweden; 3 Department of Addiction and Dependency, Sahlgrenska University Hospital, Region Västra Götaland, Journalvägen, 5 416 85 Gothenburg, Sweden

**Keywords:** Hepatitis C virus, Drug use, Model of care, Testing, Treatment

## Abstract

**Background:**

Chronic infection with the hepatitis C virus (HCV) is common in people with former or current injection drug use. Among the patients in the opioid substitution treatment (OST) program in Gothenburg, Sweden, more than 50% had been infected with HCV. However, many patients did not have any follow-up for their infection and the linkage to treatment could be improved.

**Methods:**

A model of care for HCV was introduced at an OST unit in Gothenburg, Sweden, in 2017. The aim was to increase testing and linkage to HCV treatment. A nurse and a medical doctor, both specialized in infectious diseases, performed on-site testing at the OST unit with transient liver elastography (Fibroscan) to evaluate the fibrosis stage and initiated HCV treatment. This study retrospectively reviewed the patients’ medical records to assess information regarding participation in the model of care, hepatitis C status, linkage to treatment and treatment outcome.

**Results:**

Among the 225 patients enrolled in OST at baseline, 181 were still in the OST program at the end of study (December 31^st^, 2018). In total, 29 patients, most of whom did not attend the Clinic of Infectious Diseases, were referred to the model of care. By the end of study, 17 patients (100% of those treated) reached sustained virologic response. In parallel, an additional 19 patients got treatment directly at the Clinic of Infectious Diseases.

**Conclusion:**

Integrating HCV screening and examination in an OST unit successfully linked patients to treatment. However, not all patients received treatment. To reach the goal of eliminating HCV, different models of care are needed.

## Introduction

Chronic infection with the hepatitis C virus (HCV) is one of the most important causes of liver disease worldwide. The current global estimate of the total number of people infected with HCV is 56.8 million [1]. In developed countries, the most common route of transmission is via the sharing of unsterile injection equipment. A recent Swedish study showed that in patients with HCV infection and non-alcoholic liver disease, the mortality was elevated for patients with illicit substance use disorder [2]. Effective direct-acting antiviral (DAA) treatment against hepatitis C is available at present with cure rates around 95%, and more patients now have access to treatment [3–6].

People who inject drugs (PWID) are at high risk for HCV infection, especially as harm reduction services, such as needle and syringe exchange programs (NEP), are not widely available. Also, opioid substitution treatment (OST) for opioid use disorder (OUD) may be difficult to access, a treatment that is otherwise known for its effectiveness in reducing HCV risk and active drug use [7, 8]. Among people who are on treatment for OUD, present or previous injecting drug use is common. Consequently, 50–80% of these patients are anti-HCV antibody positive as a sign of past or present HCV infection [9]. Recent data from the National Registry for Swedish NEPs shows an anti-HCV prevalence of 68% of whom 37% are HCV RNA positive (Personal communication, Steering group of National Registry for Swedish NEPs).

Before the DAA era, the treatment option for HCV infection was interferon with or without the add-on of ribavirin [10]. The efficacy of treatment was 50–80% and associated with many side effects and contraindications for people using drugs. There was, and persists in some cases, a reluctance in treating a former or current PWID for several reasons: fear of suboptimal adherence, reduced tolerability due to medical status, and the risk of HCV reinfection [11]. However, there is now strong evidence of the effectiveness of DAAs in this population [12–15]. Further, several studies have specifically looked at the combination of OST and DAA-treatment [16–19].

In Sweden, there has been a steady increase in OST programs. The Swedish HCV guidelines from 2017 and onwards are more tolerant to illicit drug use during treatment, thereby lowering the threshold for treatment. Since 2017, there has also been an increase in the number of NEPs due to a change in Swedish legislation. The guidelines for HCV treatment with DAAs have been partly revised in 2020 and 2021—with a change to prioritize PWID—making detectable HCV RNA the indication for treatment, without the need for the verification of a chronic infection [20].

Despite these efforts, uptake of HCV treatment among current and former PWID in Sweden is low and there is no good estimate of the number of untreated patients. One study estimated the cumulative HCV treatment uptake among Swedish OST patients between 2014–2017 (when DAA were used) to be 28.3% [9]. This is probably mainly due to the fact that patients with an HCV infection had to be referred to a Clinic of Infectious diseases to get treatment. However, studies in both Scandinavian and other countries have since then shown a higher treatment uptake when integrating HCV care at NEPs and OST units [19, 21].

Gothenburg is known to be the “amphetamine city” of Sweden. Heroin became more available rather late, in the 1990s. The first OST unit opened in 2003 and since, two additional units have opened, together taking care of approximately 300 patients at the time of the study. Hepatitis C has been treated at the Clinic of Infectious diseases and all patients in Gothenburg and the surrounding area are referred to this clinic. Due to lack of a NEP, there was at the time of the study very little knowledge of the PWID group and of the HCV prevalence among these patients in Gothenburg. The Swedish National Board of Social Affairs and Health estimated in 2012 that the Region Västra Götaland, where Gothenburg is situated, had a prevalence of injecting drug use of 1.2 per 1000 inhabitants, equivalent to approximately 1000 persons in Gothenburg [22].

In order to eliminate hepatitis by 2030, it is crucial to reach the patients who do not show up at ordinary outpatient clinics for treatment. The settings for OST differ between countries and even within countries. Thus studies from many different OST units, under different conditions (e.g., treatment policies, distance to Clinic of Infectious diseases), are needed to inform colleagues around the world of the feasibility of such interventions.

There has only been one study performed in Swedish OSTs evaluating HCV treatment, and that was before the DAA era [23]. It concluded that treatment in this group was feasible and showed satisfactory rates of completion. Inspired by these results, a model of care based on DAA treatment was introduced in 2017 in the OST program in Gothenburg. It included assessment for HCV and liver disease in an OST unit and offered treatment with DAAs to the eligible patients. Due to funding reasons the model of care was only operating for 10 weeks.

The aim of this study was to retrospectively evaluate the model of care introduced in an OST unit in Gothenburg, Sweden. The retrospective design gave us the opportunity to present real world data not only of results from an integrated model of care but also a broader picture of HCV treatment and testing outcome in an OST unit exposed to such an intervention.

## Patients and methods

### Study setting

The Sahlgrenska University Hospital is the largest hospital in Sweden. It is situated in Gothenburg, the second largest city in Sweden. At the time of the study there was no NEP in Gothenburg and little knowledge about the PWID population in terms of drug use pattern, HCV prevalence and number of people. The NEP in Gothenburg opened December 6, 2018. As of 2022/12/31 the HCV RNA prevalence among its 1,100 registered visitors was 22% (Personal communication, Steering group of National Registry for Swedish NEPs).

The Department of Addiction and Dependency offers both inpatient and outpatient care for persons with substance use disorder with or without psychiatric comorbidity. Moreover, the OST unit offers both medical and psychosocial treatment to patients with OUD. The medical treatment consists of either buprenorphine or methadone, the former being the most common prescription (80% of the patients). At the time of this study, the OST unit had three different outpatient clinics. Two of them were included in this study. The first is located at the hospital and targets patients with social problems and psychiatric comorbidities. It is also geographically closest to the Clinic of Infectious Diseases. The second OST unit is situated in downtown Gothenburg and the patients are more socially stable, with many of them being employed. At the time of study the OST units in Gothenburg reached approximately 300 patients, whereas the total number of patients in OST in Sweden was estimated to 2,739 [9].

The Clinic of Infectious Diseases, Gothenburg, is responsible for specialized care of infectious diseases, including blood borne viruses such as hepatitis B virus, hepatis C virus and HIV. Patients are referred to this clinic for follow-up. Despite being located less than 100 m from the hospital-based OST unit, many OST patients did not attend follow-up for their HCV infection. No outreach programs existed at the time and HCV treatment was only available on prescription from a specialist in Infectious diseases, after patient visit to the clinic.

### Standard treatment

In order to get HCV treatment, the patient needed a referral to the Clinic of Infectious diseases, after a positive HCV RNA test. First the patient got an appointment with a nurse who performed a Fibroscan. Then a new appointment is booked with a doctor. Usually treatment was not started immediately after that visit, rather the patient was discussed at a specific “treatment board”. Patients with former or current drug use might have to wait and come to a new appointment to show motivation for treatment. After two to three missed appointments, the patient was removed from the waiting-list and a new referral from a medical doctor was needed. This was the case for many of the patients in the OST program before introducing the model of care.

### Study design and sample

To increase access to HCV treatment, a model of care with integrated management of HCV infection in the hospital-based OST unit was introduced. The authors received a grant during 2017, to try out a model for testing and treating HCV in the OST unit. It was a collaboration between the Clinic of Infectious Diseases and the Department of Addiction and Dependency.

The model was simple and included three parts:

1. Testing for HCV was performed at the OST units.
2. Patients who were HCV RNA positive were offered on-site evaluation with liver elastography and additional blood screen, including the assessment of liver function.
3. Treatment was offered and administered at the OST units.

The first part lasted until September 2017. Information on HCV and treatment was given to the staff at the two OST units on multiple occasions. There was a great interest in the project. The rate of testing was initially low but increased when a coordinating nurse was appointed. For patients from the downtown OST clinic, good routines for testing were already in place, with an appointed nurse responsible. The normal routine is to test patients on OST for HCV at least once a year. In practice this routine is not followed, and some patients did not even have a single valid test registered in the electronic database. The goal was to offer a test to all patients at least once during the study period. The appointed nurse in each OST was responsible for testing and reporting of results. All blood tests were sent to the hospital laboratory for analysis.

During the second part of the project, from October to December 2017, a nurse specialized in infectious diseases brought a Fibroscan instrument once a week to the hospital-based OST unit where she performed a liver elastography exam to evaluate the fibrosis stage and took the blood tests considered necessary to start the hepatitis C treatment. The following week, when the test results were available, a physician, a specialist in infectious diseases, met with the patients and discussed the results and possible treatment. Patients were included in the model of care if they had a positive HCV RNA test and showed an interest in HCV treatment. Due to restrictions in DAA treatment at the time, based on the fibrosis stage, most patients assessed could not start treatment until January 2018, when the restrictions were lifted.

The third part of the project consisted of treatment and follow-up of patients assessed in the second part. This period extended for more than a year as treatments were going on continuously during 2018 and onward.

The present study is a retrospective cohort study comparing HCV status at baseline and at end of study for the two OST units using medical records. Baseline data was gathered at the start of the project for the hospital-based OST unit (November 11^th^, 2016) and at the time of inclusion for the downtown OST unit (July 2^nd^, 2017). The aim of the study was to evaluate the model of care in place in the OST during fall 2017. Thus the date for the end of study, December 31^st^, 2018, was chosen to allow one year for starting treatment after the end of the second part of the model of care described above. Data collection took place during 2019.

### Outcomes

- The primary outcome was the number of patients included in the model of care who were treated for HCV by 2018/12/31.
- Secondary outcomes were time to last HCV-test, number of tested patients and the total number of patients treated during the study period

### Study variables

All data was abstracted from electronic medical records. The same variables were used for both the baseline and the end of study to enable comparisons. These included age, sex, current OST treatment, antibody to HCV, HCV RNA status, if the patient attended the Clinic of Infectious Diseases, and time to most recent HCV-test. For patients participating in the model of care, fibrosis stage and HCV genotype were also assessed. A patient was included if they participated in the OST program at the date of follow-up (December 31^st^, 2018), regardless of whether the patient had been discharged from the OST unit and then got readmitted during the follow-up period.

The Swedish Ethical Review Authority approved of the study (Reference 2019–00,745/1149–18) and waived informed consent for the collection of data. The heads of the department of both clinics involved granted access to the medical records.

### Analytical approach

Statistical analysis was conducted in JMP 16.1 (SAS institute Cary, NC, 1989–2021). Binary variables were analyzed using the chi-square test and continuous variables using the Wilcoxon signed-rank test. A two-tailed *p*-value < 0.05 was considered statistically significant.

## Results

### Patient characteristics

**Fig. 1:**
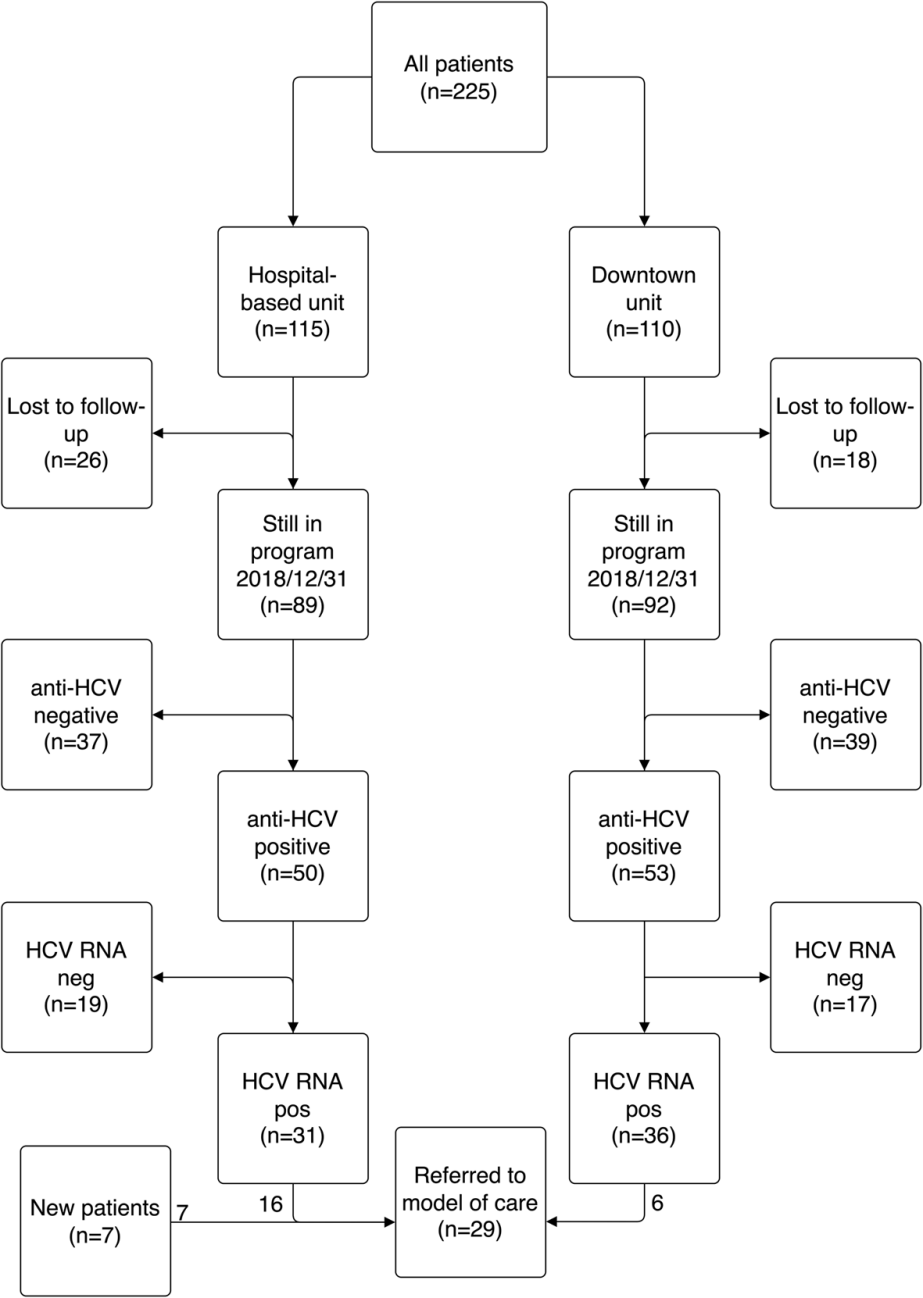
Flowchart of patients in the study. A total of 225 patients were enrolled in the OST program in Gothenburg at baseline. At the end of study on December 31^st^, 2018, 44 patients had left the program for various reasons and 29 patients had been referred to the model of care. HCV-status according to baseline data. HCV, hepatitis C virus; pos, positive; neg, negative

The patient characteristics of the total cohort are shown in Table 1. At baseline 225 patients attended the two OST units included in this study. At end of study, December 31^st^ 2018, 181 patients were still in the OST program. An overview of the study population is shown in Fig. 1 and patient characteristics of the cohort still on OST at end of study is presented in Table 2. Those who were no longer in the OST program at the end of study were younger (mean 40.3 vs 44.5 years, *p* = 0.001), but there was no significant difference in terms of HCV status.

**Table 1:** Patient characteristics at baseline and end of study

	Overall cohort (*n* = 225)		
	Baseline	End of study	*P* value^d^
Sex (M/F)	214/11	-	
Age (Years, mean (SD))	44.5 (9.5)	-	
anti-HCV^a,b^ ( ±)	134/88	133/91	n.s
HCV RNA/Ag ( ±)	90/44	65/68	0.003
Patients attending Clinic of Infectious Diseases^c^ (yes/no)	44/46	34/31	n.s
Time to last HCV-test, years (median, IQR)	1.2 (0.3–4.0)	1.3 (0.8–1.8)	0.005

*SD* Standard deviation, *HCV* Hepatitis C virus, *Ag* Antigen, *IQR* Interquartile range, *n.s*. Not significant

^a^missing data baseline *n* = 3, end of study *n* = 1

^b^one patient was anti-hcv negative on new testing

^c^Patients who had attended a visit at the Clinic of Infectious diseases during the past year or a had new appointment scheduled the following year

^d^Time to last HCV-test—Wilcoxon signed-rank test, all other comparisons—Chi-square test

**Table 2:** Patient characteristics of patients still in OST at end of study at the different OST units

	Patients still on OST 2018/12/31 (*n* = 181)			Hospital-based unit (*n *= 89)			Downtown unit *n* = 92		
	Baseline	End of study	*p* value^d^	Baseline	End of study	*p* value^d^	Baseline	End of study	*p* value^d^
Sex (M/F)	173/8	-	-	86/3	-	-	87/5		-
Age (Years, mean (SD))	45.5 (9.7)	-	-	44.6 (9.5)	-	-	46.4 (9.8)	-	-
Treatment^a^ (buprenorphine/methadone/suboxone)	-	142/35/4		-	70/17/2	-	-	72/18/2	-
anti-HCV^b^ ( ±)	103/76	103/77	n.s	50/37	50/38	n.s	53/39	53/39	n.s
HCV RNA/Ag ( ±)	67/36	46/58	0.003	31/19	18/32	0.009	36/17	28/26	n.s
Patients attending Clinic of Infectious Diseases^c^ (yes/no)	35/32	31/15	n.s	21/10	12/6	n.s	14/22	19/9	0.009
Time to last HCV-test, years (median, IQR)	1.2 (0.3–4.0)	1.3 (0.6–1.8)	0.002	5.0 (1.0–5.0)	1.3 (0.3–1.7)	< 0.0001	0.6 (0.3–1.7)	1.3 (0.7–1.8)	n.s

*SD* Standard deviation, *HCV* Hepatitis C virus, *Ag* Antigen, *IQR* Interquartile range, *n.s.* Not significant

^a^Treatment data only assessed at End of study

^b^Missing data for two patients at baseline and for one patient at end of study

^c^Patients who had attended a visit at the Clinic of Infectious diseases during the past year or a had new appointment scheduled the following year

^d^Time to last HCV-test—Wilcoxon signed-rank test, all other comparisons—Chi-square test

### Testing

During the study period. 173 patients (77%) got tested for HCV. No patient seroconverted between baseline and end of study (99 of the 132 patients who were HCV RNA negative at baseline had a follow-up test after a total of 349.4 person years). Two patients who were anti-HCV positive at baseline tested RNA positive during the study period, which gives an incidence rate of 0.6 cases per 100 person-years. Both patients had been tested outside of the OST as they had left the program at the time.

Among patients who remained in the OST program at end of study, 103/181 patients (57%) were positive for HCV antibodies and 65% of them were positive for HCV RNA at baseline. There was a very small but significant increase in time to last HCV-test for the whole cohort (baseline median 1.2 years, end of study 1.3 years, *p* = 0.002, Table 2). However, the hospital-based unit showed a large improvement (baseline median 5.0 years, end of study 1.3 years, *p* < 0.0001). At end of study, more patients were RNA-negative, 56% vs 35% at baseline (*p* = 0.003).

### Patients referred to the model of care

**Fig. 2:**
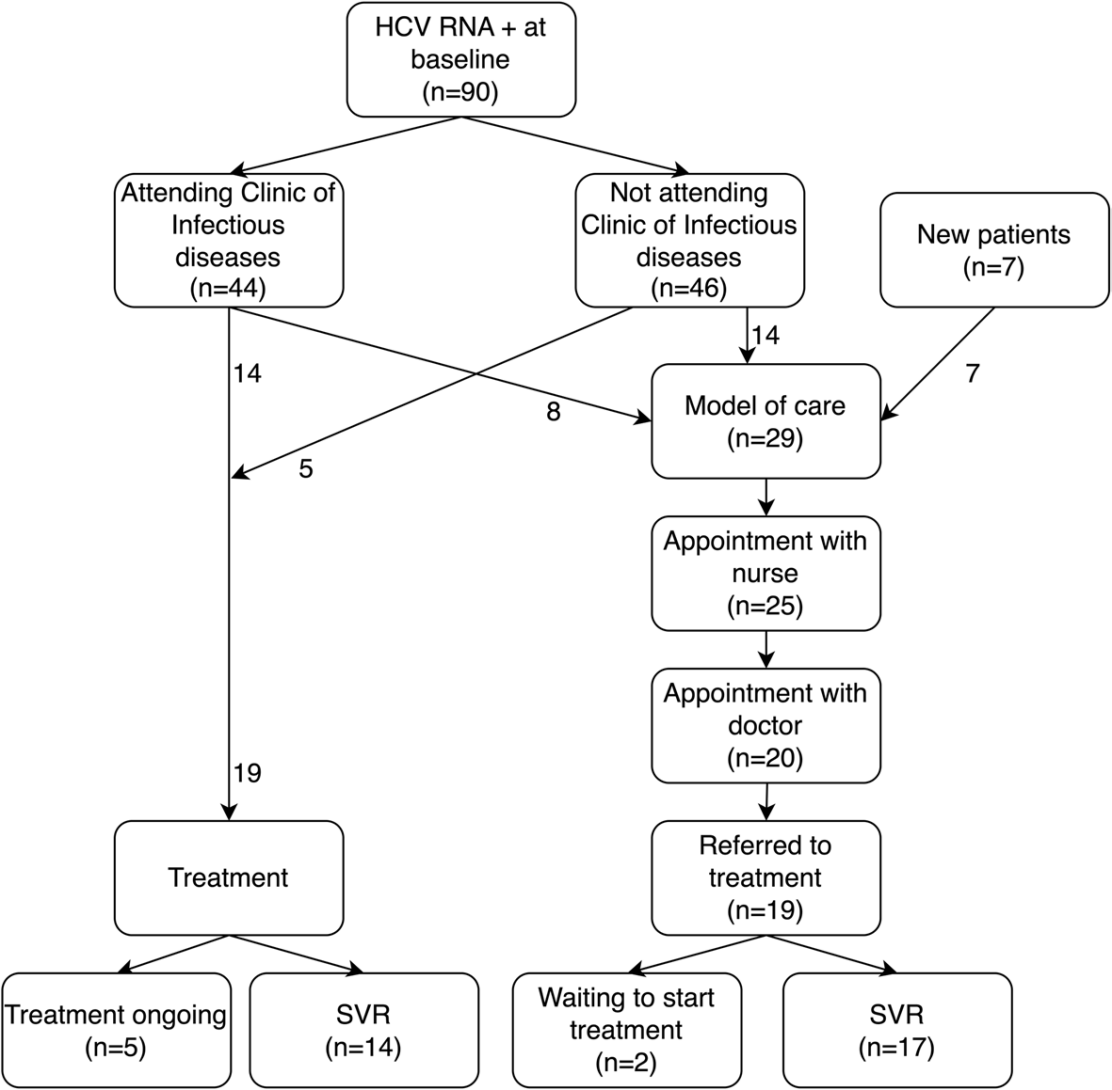
Flowchart of HCV RNA positive patients from baseline to end of study. Of the initial 90 HCV RNA positive patients, 46 did not attend the Clinic of Infectious diseases. Nineteen of these patients got treatment (14 through the model of care, 5 through standard care) whereas 22 patients who initially attended the Clinic of Infectious diseases got treatment (14 standard care, 8 model of care). An additional seven patients who started opioid substitution treatment during the study period got referred to the model of care. In the model of care 25 patients were assessed by the nurse, 20 had an appointment with the Infectious Diseases doctor, 19 intended to start treatment and 17 reached sustained virological response and were cured. HCV, hepatitis C virus; SVR, sustained virological response

**Fig. 3:**
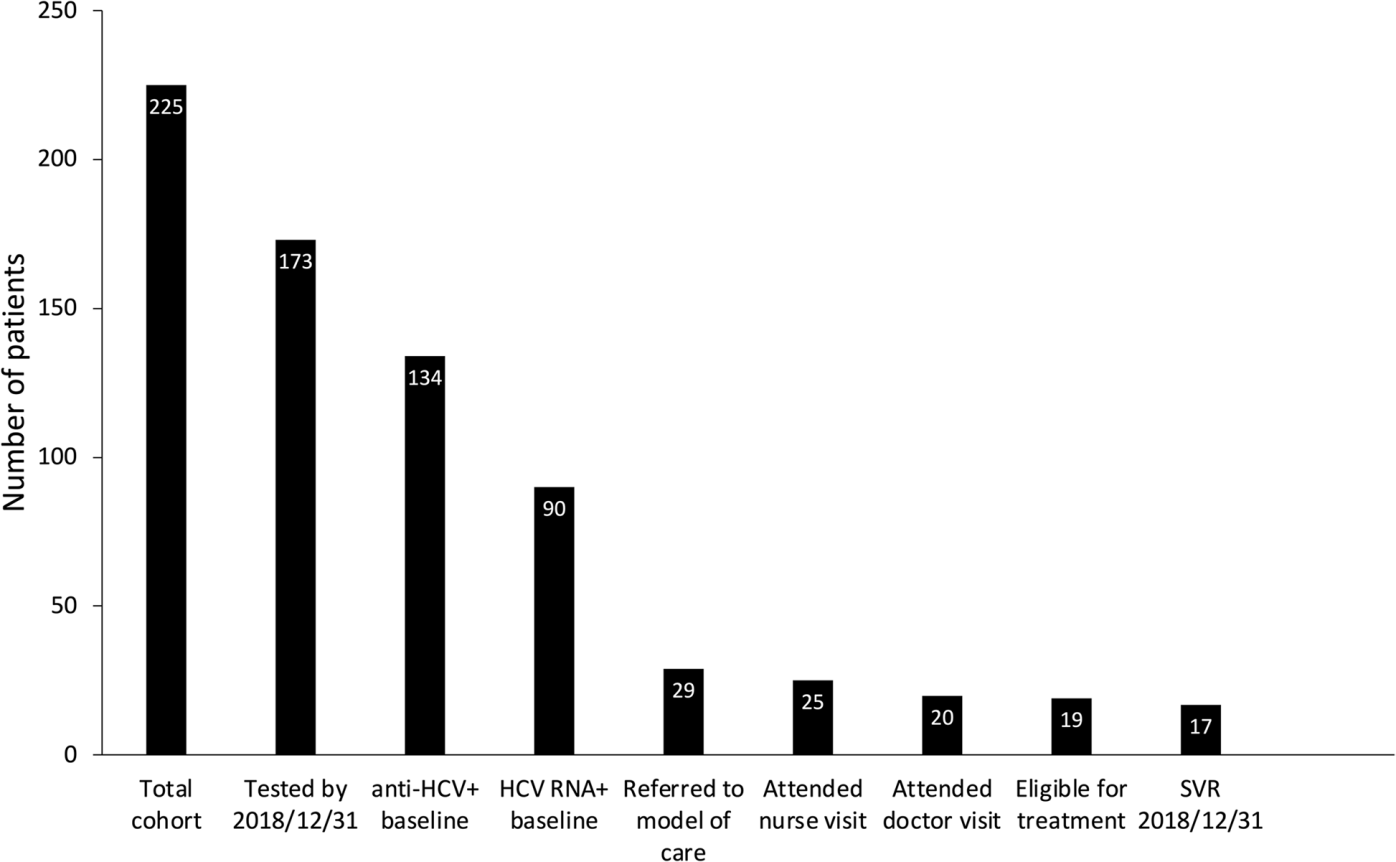
Cascade of care of the model of care evaluated in this study. The baseline cohort consisted of 225 patients. By end of study 173 of them had been tested for HCV and 90 were HCV RNA + at baseline. Out of the 29 patients referred to the model of care, 25 met the nurse, 20 saw a doctor, and 19 intended to start treatment, from which 17 reached sustained virological response (SVR) by the end of study (2018/12/31)

A total of 29 patients were referred to the model of care, of whom 22 belonged to the original cohorts (16 from the hospital-based unit and six from the downtown OST unit, Fig. 1). An additional seven patients were also included who had joined the OST program after the start of the project. Four patients did not show up for the first visit with the nurse and an additional five patients did not show up for an appointment with the doctor (Fig. 2). One patient had spontaneously cleared the HCV infection and did not need any treatment. Patient characteristics for those who at least attended the appointment with the nurse are presented in Table 3. The results of the model of care is also presented as a “cascade of care” in Fig. 3.

**Table 3:** Characteristics of patients referred to the model of care and who were assessed for hepatitis C

	Treatment (*n* = 19)	No treatment (*n* = 6)
Genotype		
1a	8	2
2	2	1
3	8	3
4	1	0
Fibroscan, fibrosis stage^a,b^		
F0-F1	10	5
F2	7	1
F3	0	0
F4	1	0

^a^One patient who received treatment had undergone a liver biopsy

^b^F0-F1 ≤ 7 kPa, F 7–9.5 kPa, F3 = 9.5–12.5 kPa, F4 =  ≥ 12.5 kPa (cirrhosis)

### Treatment

At the end of study, 19 (66%) of the patients referred to the model of care had been accepted for treatment, of whom 17 reached sustained virologic response (SVR, 100%). An additional 19 patients in the original cohort had also completed or started treatment, either because they already attended the Clinic of Infectious Diseases (*n* = 14) or got a referral from the OST to this clinic in 2018 (after the model of care was discontinued, *n* = 5, Fig. 2).

### Patients no longer in the OST program at the end of study

At the end of study, 44 patients (19.6%) were no longer in the OST program. The most common reasons for this were involuntary discharge (21 patients) and voluntary discharge (11 patients). Five patients referred to the model of care were discharged involuntarily and did not receive any HCV treatment. However, two patients, who moved to other cities, received HCV treatment and both completed the treatment at their new clinics. These two patients participated in the model of care.

## Discussion

This was a retrospective study evaluating a model of care regarding HCV assessment and treatment in two OST units in Gothenburg, Sweden. This is the first scientific evaluation of a model of care of integrated HCV management in the DAA-era in the OST units in Sweden.

As a result of this model of care, testing for HCV increased in the OST units and additional patients got referred for HCV treatment even after discontinuation of the model of care. We believe that the model of care thus had positive effects beyond the primary aim: to test and treat patients in the OST unit.

At the end of study, 17 (59%) of 29 patients referred to the model of care had reached SVR. Thus, of those who started treatment, 100% reached SVR. This can be compared with the results of a previous study in Swedish OST programs, including Gothenburg, during the interferon era [23]. Of 41 patients who started treatment, only 19 (46%) reached SVR. Reasons for stopping prematurely in that study included adverse events and low effectiveness of the treatment. The advantages of DAA treatment have opened new possibilities in the endeavour to eliminate Hepatitis C and several studies have now confirmed that SVR can be reached even in patients formerly designated as “difficult to treat” [18, 21, 24, 25]. Interestingly, a recent Finnish study found, during retrospective review of medical records, that of 20 OST patients receiving DAA treatment, 17 reached SVR12, a figure very similar to our findings. [26]

The main purpose of the model of care was to link patients to HCV treatment. It was already at that time known that offering treatment with DAAs in OST units was feasible and effective [27]. Since then, several studies have come to the same conclusion [16–18]. Different approaches have been used in OST programs in Sweden to increase the treatment uptake for HCV patients but so far, no study has shown the effectiveness of these efforts. In our cohort, 17 patients who were assessed for HCV and liver disease on-site successfully completed the treatment. A minority of them already attended the Clinic of Infectious Diseases but they had not yet received treatment and most of them did not have any follow-up for their infection at all. Probably the possibility to get tested and treated in the OST increased the number of treated patients.

The prevalence of hepatitis C is high among patients in the OST units [9]. Awareness of HCV status among patients and caregivers is a crucial first step in the cascade of care for HCV patients [28]. In Sweden, the rate of diagnosed cases of HCV is high compared to other countries, with an estimated 84% of patients diagnosed [29]. However, in a high-risk population, such as the patients in an OST unit with former or current illicit drug use, repeated testing is necessary as reinfections may occur and some patients may also clear the infection spontaneously. At the baseline of this study, 60% of patients were HCV antibody positive, and of those 67% had an active infection, i.e., 40% of the tested patients had an active and treatable HCV infection. This is in line with previous findings in other countries [9, 30].

There was a significant decrease in the time to most recent HCV test in those who remained in the program in the hospital-based unit where the model of care was implemented. An important reason for this improvement is probably due to the appointment of a coordinating nurse, underlining the importance of awareness of the risk of hepatitis C among OST patients and staff and the maintenance of routines for HCV testing. No patient had seroconverted at end of study, however two patients had been reinfected, while not on the OST program. The incidence rate in our cohort (0.6 cases per 100 person-years) was significantly lower than in a recent large prospective study of OST programs in Germany (2.5 cases per 100 person-years) [31]. Previous research has also shown that OST programs are effective in preventing HCV transmission, with incidence rates significantly lower in patients on OST compared with those not on OST [7, 8]. Our incidence rate being lower than in previous studies might indicate that the OST program in Gothenburg did not target the same population, i.e., excluding many patients with recent injecting drug use. A study from the NEP in Stockholm showed an incidence rate of 22 per 100 person-years among their participants [32]

Usually, several healthcare visits are needed before the start of the treatment and this leads to more patients dropping out. During the implementation of the model of care at the OST program of Gothenburg, restrictions were still in place for DAA treatment. Only patients with fibrosis stage 2 or above were eligible, and most of the patients in this study had to wait until the next year before receiving treatment due to these restrictions. Had treatment initiation been possible closer to the day the patient met the doctor, it might have increased the number of patients who reached SVR. This is supported by a large study of integrated HCV treatment in OST and addiction units in two Norwegian cities, that started during the time of our model of care [19]. They found a treatment initiation of 98% in the study group as compared with 77% in the standard of care group. Time to treatment initiation was also reduced to half.

More patients at the hospital-based OST unit got treatment through this model of care than those attending the unit downtown. Probably this was because they could combine their visit to the OST with the treatment for HCV. The concept of a “one-stop shop” for HCV testing and treatment, i.e., integrating these services with addiction care, has shown good results in previous studies [33–35]. Other ways of increasing access to HCV treatment is the use of mobile clinics [36, 37]. In 2022, the Swedish national plan for HCV elimination was presented [38]. Hopefully it can pave the way for such ambitious strategies as undertaken by a region in our neighbouring country Denmark, including several different interventions (e.g., test and treat in drug treatment centres and mobile unit outreach) [39].

During 2018, an additional 19 patients in the cohort still active in the OST program at the end of study, had completed or started their treatment. Fourteen patients were already attending their annual visits at the Clinic of Infectious Diseases and did not participate in the model of care. The remaining five patients got a referral to the Clinic of Infectious Diseases, showed up at their appointments, and received treatment. We believe that this could be an effect of the model of care, i.e., higher awareness of the available treatment for the HCV infection among the personnel and patients made successful referrals more likely than before the model of care was introduced. A simplified routine for HCV treatment was also established, including all testing performed at the OST clinic as well as the distribution of treatment on-site. Taken together, the aforementioned findings indicate that a combination of different models of care is needed to increase treatment rates.

The importance of OST in combination with needle and syringe exchange programs (NEPs) to prevent the spread of HCV has been pointed out previously [40]. In this study, we found only two new cases of HCV at end of study, despite increased testing in the OST with some patients not having been tested for more than 16 years. At the time of the study, Gothenburg had not yet opened a NEP (it opened in December 2018) and the retention in OST could be interpreted as a protective factor against reinfection.

At the end of study, 44 patients had left the program for several reasons (voluntary discharge, involuntary discharge, and death). According to a recent review, retention in OST programs is highly variable [41]. In our cohort, the retention rate was 80%, which is among the highest in OST programs compared to other studies. One reason for this high rate of retention might be the strict regulation on entry and discharge effective in Sweden before the study [42]. This regulation excluded patients with continuing illicit drug use, including injecting behaviour (a group known to have a lower retention rate), from entering the program.

### Strengths and limitations

This was a real-world study retrospectively evaluating a model of care implemented in the clinical practice in an OST unit. It may serve as inspiration on how to achieve HCV elimination in patients on OST in other settings with similar conditions. Our study has several limitations. No control group was available. Therefore, we cannot rule out that some or all of the patients who got treatment through this model of care would have been treated anyway. Restrictions for DAAs were lifted on January 1^st^, 2018, which could have affected the willingness to attend the visits at the Clinic of Infectious Diseases in a positive way. Moreover, the increased focus on HCV testing and the presence of a specialist in Infectious Diseases in the OST probably increased the awareness of HCV among the patients and personnel. Furthermore, our study comprised mostly male participants. This was due to the fact that most female patients attended another OST unit, not included in the model of care. Thus, we cannot draw any conclusion on potential differences in treatment uptake between male and female patients. Also, other demographic characteristics (e.g., ethnicity or social class) were not recorded in the medical records. Probably many PWID with ongoing injection drug use were excluded from the OST program due to the regulations and clinical practice in place at the time of the study. This might explain the low incidence of HCV infection. Finally, the generalisability of the results in this study is limited due to several reasons such as the partial restrictions on DAA treatment present in Sweden at the time of the model of care, different criteria for entering OST and accessibility of HCV treatment (i.e. from other providers than Clinics of Infectious Diseases) in other countries. However, this could also be a strength as many regions still struggle with these kind of restrictions and the results of this study might prove useful in efforts to eliminate HCV in these settings.

Despite these limitations we believe that the results of this study could be of importance when planning to implement integrated models of care in OST units. Our real-world data shows that patients on OST constitute a dynamic group where not one single solution is sufficient to get as many patients as possible in treatment. Our model of care was only operating half a day every week, during ten weeks, indicating that even smaller interventions can have impact on HCV testing and treatment. This can be encouraging for others who want to increase treatment uptake in their OST units.

## Conclusion

This study evaluated a model of care for integrated HCV assessment and treatment in an OST unit. HCV RNA-positive patients, the majority without current follow-up at a Clinic of Infectious Diseases, were successfully treated at an SVR of 100%. Our results are in line with previous research highlighting the effectiveness of integrated HCV care in OST units and show that this is also feasible in the Swedish setting. Despite being a time-limited project, the model of care paved the way for improved routines regarding HCV testing and treatment in the OST. To reach the goal of eliminating HCV, it is crucial to also reach other people who use drugs who are not attending OST. This is especially important in Gothenburg, where amphetamine is the most injected drug. NEPs would be a good place to reach this population as well.

## Data Availability

The participants of this study did not give written consent for their data to be shared publicly, so due to the sensitive nature of the research supporting data is not available.

## Abbreviations

HCV: Hepatitis C virus
DAA: Direct acting antiviral
NEP: Needle exchange program
OST: Opioid substitution treatment
OUD: Opioid use disorder
PWID: People who inject drugs
SVR: Sustained virological response

## References

[1] Blach S, Terrault NA, Tacke F, Gamkrelidze I, Craxi A, Tanaka J (2022). Global change in hepatitis C virus prevalence and cascade of care between 2015 and 2020: a modelling study. Lancet Gastroenterol Hepatol Elsevier.

[2] Kåberg M, Larsson SB, Jerkeman A, Nystedt A, Duberg A-S, Kövamees J, et al. High risk of non-alcoholic liver disease mortality in patients with chronic hepatitis C with illicit substance use disorder. Scand J Gastroenterol. 2020;55:574–80. 10.1080/00365521.2020.1754456 32356496

[3] Falade-Nwulia O, Suarez-Cuervo C, Nelson DR, Fried MW, Segal JB, Sulkowski MS (2017). Oral Direct-Acting Agent Therapy for Hepatitis C Virus Infection: A Systematic Review. Ann Intern Med.

[4] Feld JJ, Jacobson IM, Hézode C, Asselah T, Ruane PJ, Gruener N (2015). Sofosbuvir and Velpatasvir for HCV Genotype 1, 2, 4, 5, and 6 Infection. N Engl J Med.

[5] Forns X, Lee SS, Valdes J, Lens S, Ghalib R, Aguilar H (2017). Glecaprevir plus pibrentasvir for chronic hepatitis C virus genotype 1, 2, 4, 5, or 6 infection in adults with compensated cirrhosis (EXPEDITION-1): a single-arm, open-label, multicentre phase 3 trial. Lancet Infect Dis.

[6] Wyles D, Poordad F, Wang S, Alric L, Felizarta F, Kwo PY (2018). Glecaprevir/pibrentasvir for hepatitis C virus genotype 3 patients with cirrhosis and/or prior treatment experience: A partially randomized phase 3 clinical trial. Hepatology.

[7] Nolan S, Dias Lima V, Fairbairn N, Kerr T, Montaner J, Grebely J (2014). The impact of methadone maintenance therapy on hepatitis C incidence among illicit drug users. Addiction.

[8] Tsui JI, Evans JL, Lum PJ, Hahn JA, Page K (2014). Association of Opioid Agonist Therapy With Lower Incidence of Hepatitis C Virus Infection in Young Adult Injection Drug Users. JAMA Intern Med.

[9] Aas CF, Vold JH, Skurtveit S, Odsbu I, Chalabianloo F, Lim AG (2020). Uptake and predictors of direct-acting antiviral treatment for hepatitis C among people receiving opioid agonist therapy in Sweden and Norway: a drug utilization study from 2014 to 2017. Subst Abuse Treat Prev Policy.

[10] Flori N, Funakoshi N, Duny Y, Valats J-C, Bismuth M, Christophorou D (2013). Pegylated Interferon-α2a and Ribavirin versus Pegylated Interferon-α2b and Ribavirin in Chronic Hepatitis C. Drugs.

[11] Asher AK, Portillo CJ, Cooper BA, Dawson-Rose C, Vlahov D, Page KA. Clinicians’ Views of Hepatitis C Virus Treatment Candidacy With Direct-Acting Antiviral Regimens for People Who Inject Drugs. Subst Use Misuse. 2016;51:1218–23. 10.3109/10826084.2016.1161054 PMC6907073 27219274

[12] Dhiman RK, Grover GS, Premkumar M, Roy A, Taneja S, Duseja A (2021). Outcomes of Real-World Integrated HCV Microelimination for People Who Inject Drugs: An expansion of the Punjab Model. EClinicalMedicine.

[13] Graf C, Mücke MM, Dultz G, Peiffer K-H, Kubesch A, Ingiliz P (2020). Efficacy of Direct-acting Antivirals for Chronic Hepatitis C Virus Infection in People Who Inject Drugs or Receive Opioid Substitution Therapy: A Systematic Review and Meta-analysis. Clin Infect Dis.

[14] Grebely J, Dalgard O, Conway B, Cunningham EB, Bruggmann P, Hajarizadeh B (2018). Sofosbuvir and velpatasvir for hepatitis C virus infection in people with recent injection drug use (SIMPLIFY): an open-label, single-arm, phase 4, multicentre trial. Lancet Gastroenterol Hepatol.

[15] Norton BL, Akiyama MJ, Agyemang L, Heo M, Pericot-Valverde I-, Litwin AH. Low Adherence Achieves High HCV Cure Rates Among People Who Inject Drugs Treated With Direct-Acting Antiviral Agents. Open Forum Infect Dis. 2020;7:ofaa377. 10.1093/ofid/ofaa377 PMC7590860 33134406

[16] Akiyama MJ, Norton BL, Arnsten JH, Agyemang L, Heo M, Litwin AH (2019). Intensive Models of Hepatitis C Care for People Who Inject Drugs Receiving Opioid Agonist Therapy: A Randomized Controlled Trial. Ann Intern Med.

[17] Gutkind S, Schackman BR, Morgan JR, Leff JA, Agyemang L, Murphy SM (2020). Cost-effectiveness of Hepatitis C Virus Treatment Models for People Who Inject Drugs in Opioid Agonist Treatment Programs. Clin Infect Dis.

[18] Schmidbauer C, Schubert R, Schütz A, Schwanke C, Luhn J, Gutic E, et al. Directly observed therapy for HCV with glecaprevir/pibrentasvir alongside opioid substitution in people who inject drugs-First real world data from Austria. PLoS ONE. 2020;15:e0229239. 10.1371/journal.pone.0229239 PMC7064180 32155165

[19] Fadnes LT, Aas CF, Vold JH, Leiva RA, Ohldieck C, Chalabianloo F, et al. Integrated treatment of hepatitis C virus infection among people who inject drugs: A multicenter randomized controlled trial (INTRO-HCV). PLoS Med. 2021;18:e1003653. 10.1371/journal.pmed.1003653 PMC8205181 34061883

[20] Referensgruppen Antiviral Terapi. Läkemedelsbehandling av hepatit C-virusinfektion hos vuxna och barn 2017 - Behandlingsrekommendation. Svenska Läkaresällskapet. Available from: https://www.sls.se/globalassets/rav/rekommendationer/hcv_rekommendation_v210930.pdf. Accessed 11 Mar 2023.

[21] Blomé MA, Bråbäck M, Alsterberg S, Jerkeman A (2021). Hepatitis C treatment at a Swedish needle exchange program, a successful model of care – the ACTIONNE study. Int J Drug Policy.

[22] The National Board of Health and Welfare. En uppskattning av omfattningen av injektionsmissbruket i Sverige. Stockholm: Socialstyrelsen; 2013.

[23] Jerkeman A, Norkrans G, Lidman C, Westin J, Lagging M, Frimand J, et al. Treatment for chronic hepatitis C in a cohort of opiate substitution therapy recipients in three Swedish cities - completion rates and efficacy. Eur J Gastroenterol Hepatol. 2014;26:523–31. 10.1097/MEG.0000000000000076 24637496

[24] Beer L, Inglis S, Malaguti A, Byrne C, Sharkey C, Robinson E (2022). Randomized clinical trial: Direct-acting antivirals as treatment for hepatitis C in people who inject drugs: Delivered in needle and syringe programs via directly observed therapy versus fortnightly collection. J Viral Hepatitis.

[25] Gibbs D, Price O, Grebely J, Larney S, Sutherland R, Read P (2021). Hepatitis C virus cascade of care among people who inject drugs in Australia: Factors associated with testing and treatment in a universal healthcare system. Drug Alcohol Depend.

[26] Häkkinen M, Tourunen J, Pitkänen T, Simojoki K, Vuoti S (2022). Integrated care model and point of care diagnostics facilitate Hepatitis C treatment among patients receiving opioid agonist therapy: a retrospective review of medical records. Subst Abuse Treat Prev Policy.

[27] Butner JL, Gupta N, Fabian C, Henry S, Shi JM, Tetrault JM (2017). Onsite treatment of HCV infection with direct acting antivirals within an opioid treatment program. J Subst Abuse Treat.

[28] Denniston MM, Klevens RM, McQuillan GM, Jiles RB (2012). Awareness of infection, knowledge of hepatitis C, and medical follow-up among individuals testing positive for hepatitis C: National Health and Nutrition Examination Survey 2001–2008. Hepatology.

[29] Blach S, Blomé M, Duberg A-S, Jerkeman A, Kåberg M, Klasa P-E (2021). Hepatitis C elimination in Sweden: Progress, challenges and opportunities for growth in the time of COVID-19. Liver Int.

[30] Butler K, Day C, Dietze P, Bruno R, Alati R, Burns L (2015). The Potential Reach of Opioid Substitution Settings to Deliver HCV Care to People Who Inject Drugs in Australia. J Subst Abuse Treat.

[31] Schulte B, Schmidt CS, Strada L, Rosenkranz M, Schäfer I, Verthein U (2020). Hepatitis C Virus Prevalence and Incidence in a Large Nationwide Sample of Patients in Opioid Substitution Treatment in Germany: A Prospective Cohort Study. Clin Infect Dis.

[32] Kåberg M, Navér G, Hammarberg A, Weiland O (2018). Incidence and spontaneous clearance of hepatitis C virus (HCV) in people who inject drugs at the Stockholm Needle Exchange—Importance for HCV elimination. J Viral Hepatitis.

[33] Alavi M, Poustchi H, Merat S, Kaveh-ei S, Rahimi-Movaghar A, Shadloo B (2019). An intervention to improve HCV testing, linkage to care, and treatment among people who use drugs in Tehran, Iran: The ENHANCE study. Int J Drug Policy.

[34] Draper BL, Htay H, Pedrana A, Yee WL, Howell J, Pyone Kyi K (2021). Outcomes of the CT2 study: A ‘one-stop-shop’ for community-based hepatitis C testing and treatment in Yangon. Myanmar Liver International.

[35] Williams B, Howell J, Doyle J, Thompson AJ, Draper B, Layton C (2019). Point-of-care hepatitis C testing from needle and syringe programs: An Australian feasibility study. Int J Drug Policy.

[36] Hannula R, Söderholm J, Svendsen T, Skaland M, Nordbø SA, Steinum H, et al. Hepatitis C outreach project and cross-sectional epidemiology in high-risk populations in Trondheim, Norway. Ther Adv Infect Dis. 2021;8:20499361211053930. 10.1177/20499361211053929 PMC8558792 34733508

[37] Lazarus JV, Øvrehus  A, Demant J, Krohn-Dehli L, Weis N (2020). The Copenhagen test and treat hepatitis C in a mobile clinic study: a protocol for an intervention study to enhance the HCV cascade of care for people who inject drugs (T’N’T HepC). BMJ Open.

[38] Nationellt programområde infektionssjukdomar. Nationell elimineringsplan - Plan för eliminering av hepatit C. Sveriges regioner i samverkan; 2022. Available from: https://kunskapsstyrningvard.se/download/18.31c7bff8182e8f4deddb8b76/1663316858243/Hepatit-C-elimineringsplan-220915.pdf. Accessed 11 Mar 2023.

[39] Dröse S, Øvrehus ALH, Holm DK, Madsen LW, Mössner BK, Søholm J (2022). A multi-level intervention to eliminate hepatitis C from the Region of Southern Denmark: the C-Free-South project. BMC Infect Dis.

[40] Platt L, Minozzi S, Reed J, Vickerman P, Hagan H, French C (2018). Needle and syringe programmes and opioid substitution therapy for preventing HCV transmission among people who inject drugs: findings from a Cochrane Review and meta-analysis. Addiction.

[41] Klimas J, Hamilton M-A, Gorfinkel L, Adam A, Cullen W, Wood E (2021). Retention in opioid agonist treatment: a rapid review and meta-analysis comparing observational studies and randomized controlled trials. Syst Rev.

[42] Swedish health authority. Swedish guidelines for opioid substitution treatment (HSLF-FS 2016:1 Socialstyrelsens föreskrifter och allmänna råd om läkemedelsassisterad behandling vid opioidberoende). Socialstyrelsen. Available from: https://www.socialstyrelsen.se/kunskapsstod-och-regler/regler-och-riktlinjer/foreskrifter-och-allmanna-rad/konsoliderade-foreskrifter/20161-om-lakemedelsassisterad-behandling-vid-opioidberoende/. Accessed 11 Mar 2023.

