# Correction to: Linkage to hepatitis C treatment in two opioid substitution treatment units in Gothenburg, Sweden: a retrospective cohort study

**DOI:** 10.1186/s13011-023-00555-w

**Published:** 2023-08-03

**Authors:** Magdalena Ydreborg, Emil Lundström, Rosanna Kolleby, Sofia Lexén, Elena Pizarro, Jessica Lindgren, Rune Wejstål, Simon B. Larsson

**Affiliations:** 1 Department of Infectious Diseases, Sahlgrenska Academy, University of Gothenburg, Box 480, Gothenburg, 405 30 Sweden; 2 Clinic of Infectious Diseases, Sahlgrenska University Hospital, Region Västra Götaland, Journalvägen, 10, Gothenburg, 416 50 Sweden; 3 Department of Addiction and Dependency, Sahlgrenska University Hospital, Region Västra Götaland, Journalvägen, Gothenburg, 5 416 85 Sweden

Following publication of the original article [1], we have been notified that an error was introduced in Table 2 during the production process. The table was not aligned correctly.

The original article [1] has been updated.
